# Review: magnetic resonance imaging of male/female differences in human adolescent brain anatomy

**DOI:** 10.1186/2042-6410-3-19

**Published:** 2012-08-21

**Authors:** Jay N Giedd, Armin Raznahan, Kathryn L Mills, Rhoshel K Lenroot

**Affiliations:** 1 Child Psychiatry Branch, Brain Imaging Unit, National Institute of Mental Health, National Institutes of Health, 10 Center Drive, MSC 1367, Building 10, Room 4 C110, Bethesda, MD, 20892, USA; 2 Department of Psychiatry, University of New South Wales, Sydney, NSW, Australia; 3 Institute of Cognitive Neuroscience, University College London, London, UK

**Keywords:** MRI, Brain, Development, Sex differences

## Abstract

Improvements in neuroimaging technologies, and greater access to their use, have generated a plethora of data regarding male/female differences in the developing brain. Examination of these differences may shed light on the pathophysiology of the many illnesses that differ between the sexes and ultimately lead to more effective interventions. In this review, we attempt to synthesize the anatomic magnetic resonance imaging (MRI) literature of male/female brain differences with emphasis on studies encompassing adolescence – a time of divergence in physical and behavioral characteristics. Across all ages total brain size is consistently reported to be about 10% larger in males. Structures commonly reported to be different between sexes include the caudate nucleus, amygdala, hippocampus, and cerebellum – all noted to have a relatively high density of sex steroid receptors. The direction and magnitude of reported brain differences depends on the methodology of data acquisition and analysis, whether and how the subcomponents are adjusted for the total brain volume difference, and the age of the participants in the studies. Longitudinal studies indicate regional cortical gray matter volumes follow inverted U shaped developmental trajectories with peak size occurring one to three years earlier in females. Cortical gray matter differences are modulated by androgen receptor genotyope and by circulating levels of hormones. White matter volumes increase throughout childhood and adolescence in both sexes but more rapidly in adolescent males resulting in an expanding magnitude of sex differences from childhood to adulthood.

## Review

### Introduction

The study of male/female differences in brain and behavior is one of the most prominent, enduring, and controversial themes in neuroscience. A compelling reason to investigate sex differences in the brain is that for almost all neuropsychiatric illnesses the onset, prevalence, and symptomatology is different between males and females. For instance, Autism is between 4 and 7 times more common in boys than girls (with the prevalence discrepancy increasing with severity) [1], although a recent epidemiological study of 55,266 South Korean Youth indicates that for Autism Spectrum Disorder (ASD) the ratio is 2.5:1 male and that many females with ASD are unrecognized and untreated [2]. Attention Deficit and Hyperactivity Disorder (ADHD) and Oppositional Defiant Disorder are also significantly more common in males [3-6]. By contrast, anxiety and depression are more common in females, but this difference does not appear until puberty [7,8]. Eating disorders also typically become more prevalent in adolescence, with a markedly higher incidence in females [9,10]. Understanding the biology of male and female brain development in health and illness may provide important clues as to the mechanisms of disease and eventually guide more effective interventions. Male/female brain differences are also suggested by group average differences on a variety of cognitive/behavioral measures in typically developing children and adolescents [11]. In this report we examine anatomic differences in male and female brain development as assessed by magnetic resonance imaging (MRI).

### Total brain size and allometry

The most consistent finding in studies of brain sexual dimorphism is that the male brain is approximately 10% larger. This is supported by postmortem data [12-14] as well as *in vivo* imaging studies of adults [15-19] and children [20,21]. As the magnitude of the discrepancy is similar to that for body size, it is sometimes assumed that the difference simply reflects an overall scaling effect. However, body size alone does not account for all of the difference. Although there is a fairly strong body size/brain size correlation across species [22], within humans the correlation is not high. Also, developmental trajectories of brain and body are quite distinct. The 10% larger male brain persists from birth throughout life - even from ages 11 to 13 [23] when girls, because of their earlier growth spurt, are on an average slightly taller than boys of the same age [24]. MRI studies of adults have also demonstrated the persistence of male/female differences after accounting for body size [14,25-27].

Many of the apparent discrepancies of brain sexual dimorphism in the literature relate to whether, or how, the size of subcomponents of the brain are adjusted for the ~10% difference in total brain volume. Without adjustment, the absolute size of most structures is larger in males. If adjustments are made to subcomponents (via covariation or the use of ratios to total brain volume) an entirely different list of structures, varying by sample size and age distribution, is generated.

The issue is further complicated by non-linear scaling relationships between brain size and brain proportions (i.e. allometry). The size of neurons and other fundamental components of brain anatomy are constrained by metabolic and physical considerations, so brains of different sizes will likely need to have other than a uniform enlargement of all parts [28]. For instance, in comparisons across and within species, white matter to gray matter ratios increase with enlargement of total brain volume following a 4/3 power law [29]. This phenomenon may account for reported differences of greater gray matter/white matter ratios in females [18,30]. An analysis by Leonard and colleagues designed to address this question found that both males and females with relatively smaller cerebral volumes had larger proportions of gray matter to white matter, with the relationship slightly stronger in females [31]. The potential impact of allometric principles extends well beyond gray and white matter ratios, and many of the reported sex differences of specific brain regions in the literature may reflect the effects of total brain size differences rather than distinctive cytoarchitecture [32].

When studying neurodevelopment, there are additional issues related to potential differences in rates of growth of brain regions in males and females, such that the magnitude of sex differences may vary depending on the age of the population. Developmental trajectories of brain morphometry (i.e. size by age curves) have been found to provide discriminating features not found with static measures for separating clinical groups [33] and predicting good and bad clinical outcomes [34]. Studying the developmental trajectory of the brain using longitudinal data may thus provide a clearer picture of how brain structural differences change over the course of development, and we have emphasized such studies where available.

With these allometric caveats in mind we will now address the anatomic MRI literature with respect to male/female differences of brain sub components. Because MRI primarily classifies voxels as gray matter (comprised mostly of cell bodies, dendrites, and terminal branches of axons) or white matter (comprised mostly of myelinated axons) we will organize discussion of sex differences findings based on these tissue types.

#### Sex differences in developmental trajectories gray matter

##### *Cortex*

Neuroimaging studies across a wide age span have consistently indicated 9 to 14% larger cortical gray matter (GM) volumes in males (see [35] for review), a magnitude of difference similar to that for total brain volume.

**Figure 1:**
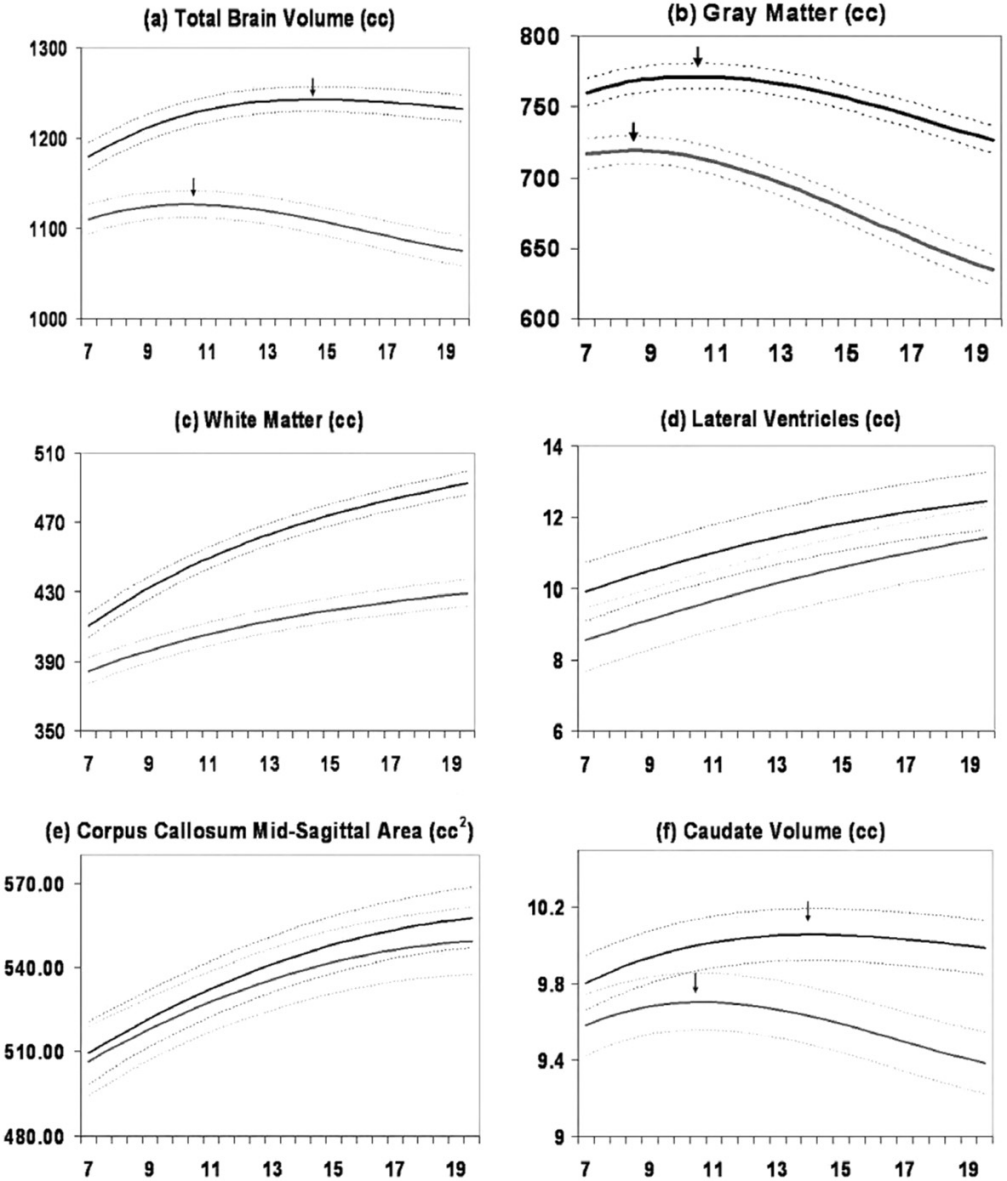
**Mean volume by age in years for males (N = 475 scans) and females (N = 354 scans).** Middle lines in each set of three lines represent mean values, and upper and lower lines represent upper and lower 95% confidence intervals. All curves differed significantly in height and shape. Figure adapted from Lenroot et al. (2007).

An analysis from an ongoing longitudinal study in our lab of 829 scans from 387 unrelated individuals (age range 3-27, 209 males), demonstrated that neurodevelopmental *trajectories* of cortical gray matter were significantly different between males and females [23]. Total brain size followed an inverted U trajectory in both sexes, with peak total brain size occurring at approximately 10.5 years in females and 14.5 years in males. Regional GM volumes also followed an inverted U shaped maturational curve and peaked earlier in females [see Figure 1.

Cortical volume is a product of surface area and cortical thickness - constituents that have distinct developmental trajectories, heritability, evolutionary histories, and relationships to pathology [36]. Recent studies have begun to separately quantify surface area and cortical thickness, with some reporting no differences [16,37,38], some reporting greater thickness in males [37], and some reporting greater thickness in females [39]. In the latter study of 60 young adults, when not adjusted for total brain volume, cortex was thicker in females in the left inferior and superior frontal gyri, and to a lesser extent in the superior pre- and post-central regions and occipital lobe, whereas males had an area of increased thickness in the left posterior temporal lobe. Using the same methodology for 176 participants ranging from 7 and 87 years of age, females, after covarying for total brain volume, had thicker cortices in the right inferior parietal and posterior temporal regions [40]. The surface area of the parietal lobe is reported to significantly decrease across development (7-50 years) in females but not males [41]. As increases in total gray matter volume are driven primarily by expanded surface area [42], issues of covariance for total brain volume are highly relevant for studies of cortical thickness. To address these possible allometric confounds, Sowell et al. [40] examined a sub sample of 36 participants (18 males and 18 females) matched on total brain volume and age. They found that, even when overall brain volume was not different, females had thicker cortex in the right lateral frontal, temporal, and parietal cortices. A similar approach was taken in an independent analysis of sex differences in 48 adults (24 male, 24 female), who were chosen from a large databank of imaging data on the basis of having identical brain volumes. This study also found that females had larger amounts of gray matter in several brain regions, including the caudate, left superior temporal gyrus, and left superior frontal gyrus [43].

### Subcortical gray matter

#### Basal ganglia

The basal ganglia are a collection of subcortical nuclei (caudate, putamen, globus pallidus, subthalamic nucleus, and substantia nigra) that are involved in circuits mediating movement, higher cognitive functions, attention, and affective states. The caudate has been reported as proportionately larger in females by several studies across different ages and using different methodologies [21,35,44,45], which is intriguing given the findings of reduced caudate volumes in male predominant disorders such as Attention Deficit Hyperactivity Disorder and Tourette's syndrome [46]. However, a proportionately larger female caudate was not seen in a recent study of 1004 young adults, which reported significantly larger globus pallidus and putamen volumes in male participants (that remained significantly different after covarying for total brain volume), but no sex differences in the caudate nucleus or nucleus accumbens [47].

#### Amygdala and hippocampus

Nonhuman primate studies indicate a relatively high number of androgen receptors in the amygdala [48] and a relatively higher number of estrogen receptors in the hippocampus [49]. This is consistent with a reports of proportionately larger amygdalae in adult men [19,50,51], and that during adolescence amygdala volume increases significantly only in males, while hippocampal volume increases significantly only in females [52]. Similarly, in a sample of 46 males and 46 females aged 8-15 years, hippocampal size was larger in girls whereas gray matter volume in the amygdala was larger in males [53]. These areas have also been associated with disorders such as depression and anxiety disorders, which show distinct differences between the sexes [54-56].

### White matter

The “white” of white matter (WM) is from the color of myelin, a fatty insulating sheath from oligodendrocytes that wraps around axons and increases conduction velocity. Given that gray matter and white matter are parts of the same neural circuitry, and robustly interact throughout the life span, it is noteworthy that they have quite different developmental trajectories. Whereas gray matter volumes generally follows an inverted U shaped trajectory with peak size occurring at different ages in different regions, white matter volumes increase throughout the first 4 decades. In the NIH longitudinal sample [23] and in an independent cohort of 188 children and adolescents [57], WM in males grew more rapidly, resulting in increasingly larger volumes relative to females with age. In contrast to the relatively stable magnitude of gray matter dimorphism across development, white matter sex differences increase from birth, through adolescence, and into adulthood [35].

#### Corpus callosum

The corpus callosum was one of the first structures in which sex differences were reported [58]. The most typically reported metric has been the sagittal area, which despite being relatively easy to quantify has been controversial in terms of the presence of sex differences. Although most studies have reported increased size in males [59,60], or minimal differences [61], it has been strongly debated whether the corpus callosum was proportionally larger in females for brain size [62,63]. Similar to the questions about relative proportions of gray and white matter, more recent analyses have suggested that the differences in the relative size of the corpus callosum are instead due to scaling issues related to overall brain volume, and that there are not significant differences between males and females once this is taken into account [31,64]. Studies of the development of the corpus callosum have also had mixed findings. The longitudinal NIH study described above did not find significant differences in the size or rate of growth of the mid-sagittal area of the corpus callosum [23]. Luders and colleagues studied development of corpus callosum thickness and shape in a cross-sectional sample of 190 children aged 5-18, and found a complex pattern of sex-specific changes with age in several regions, with an overall more rapid increase in thickness in this age range in females [65].

#### Diffusion tensor imaging

Diffusion tensor imaging (DTI) quantifies diffusion of water through different regions of the brain [66]. If unconstrained, water molecules will randomly diffuse in all directions. Non-random diffusion can be used to infer constraints place upon the motion of water by physical features such as cell membranes or interactions with large molecules [67]. Fractional anisotropy (FA) is a measure used to indicate the degree of non-randomness of the diffusion. Regions that are highly myelinated, such as the corpus callosum, have high fractional anisotropy values because water diffusion is constrained by the tightly packed unidirectional axons. Mean diffusivity (MD), the overall speed of diffusion, tends to be decreased by these same factors. As would be predicted by increasing myelination, overall FA increases and MD decreases during childhood and adolescence [68-72]. Whether the specific trajectories of these diffusion parameters are sexually dimorphic has been the topic of a handful of investigations.

In a DTI study of 52 male and 52 female adolescents higher FA and lower MD in females was found in the splenium of the corpus callosum, whereas in males FA was higher and MD lower in bilateral frontal WM regions, the right arcuate fasciculus, and left parietal and parieto-occipital WM [72]. Left frontal lobe FA was positively correlated with age in boys, but negatively correlated with age in girls. This sex by age interaction suggests that sexually dimorphic processes are at work beyond differences in maturational rates.

To further characterize the relationship between sex and white matter integrity in young adolescents (age 12-14 years), Bava and colleagues examined FA and MD in addition to two further measures of diffusivity, axial diffusivity (AD) and radial diffusivity (RD) [73]. AD is thought to reflect primarily axonal integrity or changes [74,75], whereas RD is thought to reflect changes in myelination or glial alteration [76-78]. As no sex differences were observed with RD, the authors suggest that myelination may not sufficiently characterize white matter differences between males and females. Results of their study showed that females have higher FA in the right superior corona radiata, higher FA and AD in bilateral corticospinal tract, and lower MD in the right inferior longitudinal fasciculus and left forceps major than their age-matched male counterparts. The sex differences observed in the corticospinal tract may reflect greater directional coherence and organization of white matter pathways in females. Males, on the other hand, had higher AD in the right superior longitudinal fasciculus, right inferior longitudinal fasciculus, and forceps minor, which may reflect a restriction of diffusion along these association fibers while axonal caliber increases. The findings of Bava et al show little convergence with those of Herting et al. [79] however, who also examined tract-based differences in DTI metrics between adolescent male and females; in the Herting study boys had higher FA in cortico-spinal, cortico-subcortical, and long-range association white matter tracts than girls, girls showed greater MD in frontal and temporal white matter.

Sex differences in subcortical microstructure have also been characterized using DTI [80]. In a comparison of 25 females and 25 males, microstructural differences in the thalamus, corpus callosum, and cingulum were observed, with males displaying higher FA and lower RD in these areas.

### Cerebellum

Cerebellum is Latin for “little brain” and in many ways this is an apt description. Although only about 1/9 the volume of the cerebrum, the cerebellum actually contains more brain cells than the cerebrum [81]. The functions of the cerebellum have traditionally been described as related to motor control, but it is now commonly accepted that the cerebellum is also involved in emotional processing and other higher cognitive functions that mature throughout adolescence [82,83].

In a sample of 25 males and 25 females, age 5 to 24 years, each with three or more longitudinal scans at approximately two year intervals, developmental curves of total cerebellum size follow an inverted U shaped developmental trajectory with peak size occurring at 11.8 in girls and 15.6 in boys [84]. Cerebellar volume was 10% to 13% larger in males depending on the age of comparison, and the sexual dimorphism remained significant after covarying for total brain volume. Subdivisions of the cerebellum had distinct developmental trajectories, with phylogenetically more recent regions maturing particularly late. The cerebellum's unique protracted developmental trajectories, sexual dimorphism, preferential vulnerability to environmental influences, and frequent implication in childhood onset disorders such as autism and ADHD make it a prime target for pediatric neuroimaging investigations.

### Relationship of findings to sex differences in behavior and risk for psychopathology

The relationship between reported sex differences in brain anatomy and sex differences in behavior remains unclear, and is an area of active investigation. Qualitatively, documented sex-differences in cortical gray matter [85-87],sub-cortical structures [21] and white matter tracts [73] frequently show some spatial overlap with brain systems that have been implicated in sexually dimorphic behavioral domains or disease states. Such apparent convergences are hard to interpret however for several reasons. First, since structural neuroimaging assays of sex differences, and sexually dimorphic behaviors/disease states are rarely carried out simultaneously, integration of findings across these two literatures is complicated by methodological differences between different studies. Secondly, the degree of overlap between regions of sexually dimorphic neuroanatomy and neuroanatomical correlates of sexually dimorphic behavior has yet to be assessed using statistical methods capable of controlling for the base-rate likelihood of overlap between these classes of often distributed maps. Third, the developmentally dynamic nature of sex-differences in behavior and risk for psychopathology means that any potential relationship between anatomical and behavioral variation would best be assessed using longitudinal neuroimaging methods that contrast males and females while tracking both behavior and anatomy. In addition to the many empirical gaps that prevent simple links being forged between sex-differences in anatomy and sex-differences in behavior, there are also several biologically plausible scenarios under which one would not expect convergence between these two properties of the brain.

### Relationship of findings to distribution of sex steroid receptors and hormone levels

Sexually dimorphic brain regions (e.g. basal ganglia, amygdala, hippocampus, and cerebellum) are notable as having high densities of sex steroid receptors [19,88]. The hypothalamus, consistent with its central role in reproductive function [89], is a “hub” of regions high in sex steroid receptors. Both the hypothalamus itself and areas with strong connections to it have a high density of estrogen, androgen, and progesterone receptors. Prominent among these regions are the amygdala, bed nucleus of the stria terminalis, and parts of the nucleus of the solitary tract and parabrachial nucleus. Goldstein et al. (2001) found the hypothalamus and amygdala (as well as frontomedial cortex and the angular gyrus) to be proportionately larger in men whereas frontal and medial paralimbic brain regions were proportionately larger in women. The differences with the greatest effects sizes were also areas richly endowed with sex steroid receptors during development (as determined by animal models) [19]. Intriguingly, the relationships between sex steroid levels and neuroimaging measures of sub-cortical anatomy were recently examined by Neufang et al [53], who found that the amount of gray matter in the amygdala was predicted by testosterone levels in both males and females. Testosterone levels also predicted hippocampal size in females, but with younger females having larger hippocampi.

Immunohistochemistry methods and in situ hybridization techniques are extending the range of brain regions, cell types, and cellular locations in which sex steroids have been found [90,91]. Recognition that splice variations in steroid receptor transcription may affect both function and the detection by assays [92,93] and further characterization of maturational changes in the expression of sex steroid receptors, particularly during puberty [94], will deepen explorations of hormone/brain/behavior interactions.

In a recently published study [87], we sought to directly assess the spatial convergence between the regions where sex, and sex steroid signaling modulated cortical maturation across human adolescents. Sex differences in cortical thickness varied over the course of adolescent, so that widespread thickness excess in males relative to females at age 9 years became more pronounced across adolescence in parietal regions, but gradually diminished in prefrontal areas. These shifts arose via generalized age-related cortical thickness reduction in both sexes, taking place at a relatively slower rate in males than females in posterior regions, and visa versa prefrontally. The resultant prefrontal "convergence" in cortical thickness was slowest to occur in late-maturing prefrontal sub-regions implicated in behavioral control. Within this context of sexually dimorphic cortical development, carriage of an androgen-receptor genetic variant thought to confer more efficient androgen signaling was associated with relative “masculinization" of cortical thickness maturation within each sex. These findings add to the growing body of literature which suggests that the several-fold greater levels of circulating androgens in males compared with females during prenatal and adolescent development [95,96] may contribute to between-sex differences in human cortical anatomy.

In a recent study fetal testosterone levels were found to predict gray matter volumes in boys (8-11 years) in a subset of brain regions that differed between sexes in an independent sample [97]. Regions of overlap between testosterone effect in males and regions of sex-differences in brain anatomy included the right temporoparietal junction/posterior superior temporal sulcus, which had greater gray matter volume in males compared to females, and correlated positively with fetal testosterone levels. Conversely gray matter volume in the planum temporale/parietal operculum, and posterior lateral orbitofrontal cortex was negatively correlated with fetal testosterone levels, and was greater in females compared to males. The researchers of this study noted that amygdalar and hypothalamic volumes, while not correlated with fetal testosterone levels, were greater in males compared to females. Such dissociations may reflect the multitude of non-hormonal candidate mechanisms for sex-differences in the brain including differences in sex-chromosome dosage and environmentally-determined sex-differences in experience (Arnols Ref).

The effects of testosterone have also been examined for WM development in a cross sectional MRI study of 408 healthy adolescents (204 males; age range 12-18) which included assessment of serum testosterone levels and AR genotype [98]. In addition to conventional anatomic MRI and DTI they used magnetization transfer ratio (MTR) imaging, which is more directly affected by the total amount of myelin, to further characterize WM development. Consistent with previous reports WM increased more rapidly in males. Testosterone levels did not add significantly to age in explaining variation in WM volume in the overall group. However, there was a trend toward interaction between testosterone levels and the subset of males with the AR genotype having fewer CAG repeats. Although WM volume increased, MTR decreased with age accounting for 8% of the variance in males and 1% in females. These findings raise questions about interpretations of DTI, MTR, and anatomic WM measures at molecular and cellular levels and suggest that the rapid increase in WM volume in males may be related to other structural elements such as axonal volume rather than myelination.

Sexually dimorphic effects of puberty on brain anatomy are being examined in a longitudinal study of typically developing twins in the Netherlands [99,100]. In a sample of 57 male twins (age 9.20 + - 0.10) and 47 female twins (age 9.21 + -0.12) luteinizing hormone levels (an indication of onset of puberty) were positively correlated with WM volume [99]. In an overlapping cohort of 37 males (age 11.6 + - 1.0 ) and 41 females (age = 12.2 + -1.2) effects of estradiol and testosterone levels were also examined [101]. Total GM volumes correlated negatively with estradiol levels in females and positively with testosterone levels in males.

### Limitations

The spatial resolution of standard MRI acquisitions often makes direct comparisons to post mortem data challenging. Lack of gross differences as assessed by MRI may belie important differences that occur in smaller subregions or at scales not detected by MRI. For instance, a post mortem finding of greater degree of myelin staining in the superior medullary lamina along the surface of the parahippocampal gyrus in females versus males from ages 6 to 29 years [102] is unlikely to be detected in gross volume of white matter. Another challenge is comparing studies using different inclusion/exclusion criteria on different scanners with different methods of structure quantification and different statistical analyses.

## Conclusions

Male and female brains are overwhelmingly more alike than different. The most robust difference, in both pediatric and adult studies, is a 10% larger total brain size in males. Other brain morphometric differences depend on whether or how subcomponents are adjusted for total brain volume with the largest effect sizes reported for the caudate nucleus, amygdala, and hippocampus, and cerebellum. Non-linear scaling effects may lead to regional differences attributable to variation in brain size alone [31,103].

Recognition of the importance of considering trajectories of development as opposed to average size across wide age ranges is another key issue in interpretation of sex differences in brain imaging studies. Gray matter volumes tend to follow inverted U shaped trajectories with peak size occurring earlier in females. White matter volumes become increasingly divergent as males and females reach adulthood. As brain regions growing at different rates between males and females the magnitude or even direction of the difference depends on the age at which measurements are made [21,23,51].

As cognitive/behavioral abilities are subserved by widely distributed neural networks analytic approaches such as graph theory that capture relationships amongst structures are likely to be more informative.

DTI and MTR imaging have implied sexual dimorphism of brain microstructural features such as myelination and tissue organization. Examination of hormonal, brain, and behavior changes at different pubertal stages [104,105] or phases of the menstrual cycle may provide further insight into the relationship of sex steroids and neuronal plasticity.

The goal of investigations of brain sexual dimorphism is not to declare a “winner” in some aspect of brain function. It is to elucidate mechanisms of typical and atypical brain development that may guide the search for more effective interventions.

## Abbreviations

(AR): Androgen receptor; (CT): Cortical thickness; (DTI): Diffusion tensor imaging; (FA): Fractional anisotropy; (MD): Mean Diffusivity; (AD): Axial Diffusivity; (RD): Radial Diffusivity; (fMRI): Functional MRI; (GM): Gray matter; (AR-H): High functioning androgen receptor; (AR-L): Low functioning androgen receptor; (MRI): Magnetic resonance imaging; (AR-M): Medium function androgen receptor; (SFG): Superior frontal gyri; (WM): White matter.

## Competing interests

No competing interests.

## Authors contributions

All contributed to writing and review of literature. All authors read and approved the final manuscript.
